# Wide-Band Color Imagery Restoration for RGB-NIR Single Sensor Images

**DOI:** 10.3390/s18072059

**Published:** 2018-06-27

**Authors:** Xavier Soria, Angel D. Sappa, Riad I. Hammoud

**Affiliations:** 1 Computer Vision Center, Edifici O, Campus UAB, Bellaterra, 08193 Barcelona, Spain; asappa@cvc.uab.es; 2 Facultad de Ingeniería en Electricidad y Computación, CIDIS, Escuela Superior Politécnica del Litoral, ESPOL, Campus Gustavo Galindo, Km 30.5 vía Perimetral, Guayaquil 09-01-5863, Ecuador; 3 BAE Systems FAST Labs, 600 District Avenue, Burlington, MA 01803, USA; riad.hammoud@baesystems.com

**Keywords:** RGB-NIR sensor, multispectral imaging, deep learning, CNNs

## Abstract

Multi-spectral RGB-NIR sensors have become ubiquitous in recent years. These sensors allow the visible and near-infrared spectral bands of a given scene to be captured at the same time. With such cameras, the acquired imagery has a compromised RGB color representation due to near-infrared bands (700–1100 nm) cross-talking with the visible bands (400–700 nm). This paper proposes two deep learning-based architectures to recover the full RGB color images, thus removing the NIR information from the visible bands. The proposed approaches directly restore the high-resolution RGB image by means of convolutional neural networks. They are evaluated with several outdoor images; both architectures reach a similar performance when evaluated in different scenarios and using different similarity metrics. Both of them improve the state of the art approaches.

## 1. Introduction

Color image restoration is the process applied to a given image in order to obtain color image values similar to the real ones. This color difference problem is generally generated by acquisition sensors [1]. Color image restoration is also required to solve specific problems related to compression, dehazing, deblurring and others, to name a few. There are several approaches to such a problem, that range from simple interpolation [2] to advanced deep learning based techniques [3]. The color image restoration problem also appears in the multi-spectral imaging domain. Multi-spectral images (MSIs) are obtained as the composition of multiple wavelength spectral bands (e.g., reference [4]). In the current work, we focus on MSI cameras operating in the visible (RGB) and near-infrared (NIR) sub-spectrum (400–1100 nm). The NIR spectral band goes beyond the range of the human visual system (400–700 nm), where visually similar samples may exhibit very different characteristics (e.g., [5,6,7,8]). Such properties have been largely exploited in remote sensing applications where different spectral bands are used to characterize elements, such as materials, vegetation, water pollution, etc. (e.g., [9,10]).

**Figure 1:**
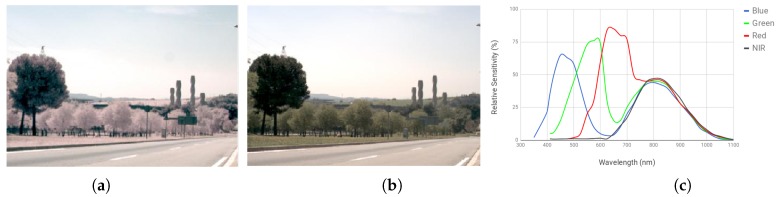
(**a**) RGB+N image (infected with near infrared, NIR); (**b**) RGB image obtained with an infrared cutoff filter (free of NIR infection); (**c**) multi-spectral sensitivity graph for a single sensor camera without an infrared cutoff filter.

Among all the different spectral bands used in the computer vision beyond the visible spectrum, the near-infrared has been the most widely explored, since, on one hand, it exhibits unique properties related to material energy absorption and reflectance. On the other hand, sensors based on silicon (SiO_2_) are sensitive to NIR up to 1100nm, hence NIR cameras are relative cheap in comparison to cameras that work with other technology, at other spectral bands. Since the NIR band is the closest in wavelength to the radiation detectable by the human eye, NIR images share several properties with visible images (see illustrations in Figure 1).

Surface reflection in the NIR band is material dependent. For instance, most dyes and pigments used for material colorization are somewhat transparent to NIR. This means that the difference in the NIR intensities is not only due to the particular color of the material, but also the absorption and reflectance of dyes [5]. The absorption/reflectance properties mentioned above are used, for instance, in remote sensing applications for crop stress (water and nutrient stress being the most common) and weed/pest infestations. These applications are based on the fact that NIR is not absorbed by any pigments within a plant. Rather, it travels through most of the leaf and interacts with the spongy mesophyll cells. This interaction causes about half of the energy to be reflected and the other half to be transmitted through the leaf. In plants with turged and healthy mesophyll cell walls and in dense canopies, more NIR energy will be reflected and less transmitted. This cell wall/air space interaction within these cells causes healthy vegetation to look very bright in the NIR spectral band. In fact, much more NIR energy is reflected than visible energy. By monitoring the amount of NIR and visible energy reflected from the plant, it is possible to determine the health of the plant.

In attempts to exploit such attractive properties (absorption/reflectance at the NIR band and low cost technology), new cameras have been developed that are able to work from the visible spectrum (400–700 nm) up to the NIR spectral band (700–1100 nm), providing the information in four independent channels—throughout this paper, this technology will be referred to as Single Sensor Cameras (SSC). [11]. Although interesting, the main problem with this technology lies in the overlap between bands, as can be seen in Figure 1. NIR sensors are sensitive to RGB spectral bands, and part of the NIR information also goes below 700 nm, generating visually distorted RGB images (in general, biased towards the red channel, see illustration in Figure 1a,b [4,11,12,13,14]). In fact, this problem is termed “spectral cross-talk” in the state-of-the-art methods . The current work is focused on this problem (spectral band overlap) and tries to restore color by removing NIR information from the R, G, B channels. There have been recent approaches in the literature based on the sensor’s model and interpolation (they will be reviewed in Section 2). Contrary to these approaches, in the current work, a RGB color restoration from a multi-spectral image (RGBN, N: NIR), based on the deep learning framework, is proposed which assumes that the sensor responses are unknown. These images are acquired by an SSC which generates a single image containing all bands (RGBN: visible and near infrared wavelength spectral bands). In summary, our main contribution in this paper is two-fold:

- To propose two custom CNN architectures to generate RGB images from their corresponding RGB+N data. The codes of these fully trained networks are available through https://github.com/xavysp/color_restorer.
- To extend our previous work [15] via reporting additional results and findings on a publicly available dataset [16]. This dataset has been enlarged through corresponding data augmentation.

The rest of the paper is organized as follows: In Section 2, a description of color restoration related approaches is given. Then, the proposed approach is presented in Section 3. Firstly, a short description of Convolutional Neural Networks is given, and then the proposed architecture and the framework used in the experiments are presented. The Experimental results are described in Section 4. Finally, conclusions and future works are presented in Section 5.

## 2. Related Works

In this section, some works related to color image restoration algorithms for multi-spectral images are briefly reviewed. Firstly, techniques based on color correction matrices and interpolations are presented. Then, deep learning based approaches for image enhancement, mainly super and high-resolution approaches, are reviewed, since they are similar to the problem tackled in the current work, especially in regard to the process that addresses the reconstruction of high resolution images and edge preservation.

Most of the approaches for multi-spectral color restoration in the literature have focused on interpolation and spectral decomposition (signal processing level) to perform the color restoration process in order to eliminate crosstalk in RGB+N images. In [11], the authors proposed an approach based on color correction methods using a color-checker chart as the calibration target. In this approach, initially, a white balance process is applied, and then chromatic adaptation is considered to finally make color corrections with a linear transformation matrix. Although interesting results were presented, it should be mentioned that this approach has been tested in a particular set of RGBN images obtained in laboratory conditions with a fluorescent lamp. The obtained color corrected images were evaluated using an X-rite color checker chart. It should be noted that in the evaluation scenario, the visible spectrum was not affected by sunlight. Furthermore, the tested scenario does not contain vegetation where the NIR influence plays a considerable role. This color correction in the constrained scenario was also considered in [17] (an extension of a former work presented in [18]), although the authors mentioned the presence of sunlight in the indoor tested scenes. In these works, the authors tackle the complex problem of restoring three channels ($R_{vis}$, $G_{vis}$ and $B_{vis}$), which are contaminated with an unknown quantity of NIR information. The authors propose a method that is based on a spectral decomposition. It implies that the spectral response of each channel will correspond to the RGBN values. Each one of the channels contains a NIR and a VIS spectrum part, which results in the following formulation: $N=NIR_{vis}+NIR_{nir}$, $R=R_{vis}+R_{nir}$ and so on. Hence, the corrected colors are obtained as follows:

(1) $${\left({\hat{R}}_{vis},{\hat{G}}_{vis},{\hat{B}}_{vis}\right)}^{T}=M\;{\left(R,G,B,N\right)}^{T},$$

where M is the decomposition matrix obtained by modeling the sensor sensitivity and spectral band correlation; it is a scaling factor coefficient that relates the visible spectrum and NIR bands. The authors describe that the additional NIR information infected in the RGB channels may be an unknown value. In other words, the spectral sensitivity curves presented in Figure 1c depend on the sensor and are needed to solve Equation (1). Note that the amount of NIR information will depend on both the light in the scene and the type of material present on it. In other words, just using a demosaicing technique with different color filter array patterns does not generate images like those obtained with an infrared-cutoff filter lens. The NIR information needs to be removed from the RGB channel [17,19].

**Figure 2:**
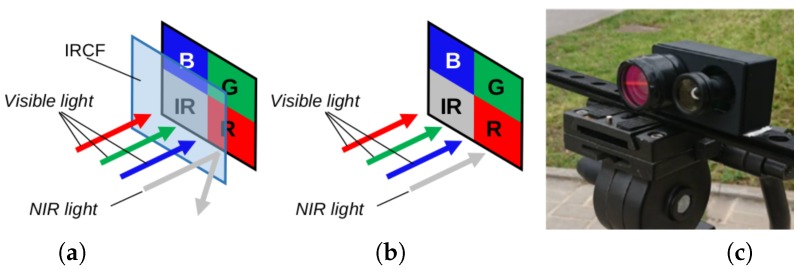
Illustration of (**a**) an infrared filtered imaging system (IRCF: infrared cut-off filter); (**b**) RGBN imaging system; (**c**) See3CAM-CU4 RGBN cameras used in the experiments. The infrared cut-off filter can be seen in the left side of the figure; it is used to generate the ground truth RGB images.

Another color image restoration technique was proposed in [13]. In this case, the visible image was obtained by subtracting a different amount of NIR information at each visible channel, according to previously computed coefficients. These coefficients are obtained from the sensor sensitivity curves (an example of such curves is presented in Figure 1c). This particular formulation is only valid for a NIR wavelength range of {650–819 nm}, since the camera used in that work was only sensible to the aforementioned range values. Although the results are quite good and the algorithm is efficient and fast to compute, its main drawback lies in the short wavelength validity as well as in the coefficient estimation, which, once estimated, are used as constant values for the whole set of images. Then, ref. [20] using different Multi-Spectral Filter Arrays (MSFA) patterns, linear mappings and demosaicing techniques, like [13], improves results for the cross-talk problem for images acquired by an SSC. In [21], the authors proposed a demosaicing and color correction matrix to improve the quality of acquired RGB and NIR images. Demosaicing refers to the process of obtaining the {$R_{vis},G_{vis},B_{vis},N_{nir}$} components for a given pixel by interpolating the information provided by the sensor which is a single square array {B,G} on top and {NIR,R} on bottom (see an illustration of this pixel composition in Figure 2). The performance of this approach has been only evaluated using indoor images; when it is used in outdoor scenarios, the color correction does not work properly so the obtained results do not look like natural colors provided by a RGB sensor camera. More recently, in [19] the authors proposed a model based on [14] for image formation that assumes the sensor response is known. Its main contribution was convolutional sparse coding (using a filter dictionary).

Nowadays, a large number of classical computer vision problems are being solved under the deep learning (DL) framework [22,23]. In the image enhancement field, several DL based approaches have been proposed [24,25], in particular, focusing on the super-resolution (SR) [24] and image denoising (ID) [26,27] problems. Although not focused on color correction, in the image enhancement domain, high quality images are intended to be obtained from the given low resolution or corrupted ones. The current work is related to these approaches in the sense that a better color image representation is sought from the given NIR infected images. Hence, in the rest of this section, ID and SR representative techniques based on the DL framework are briefly described.

A few years before the proliferation of CNN-based approaches, the authors of [28] developed an architecture based on CNN for natural image denoising. The proposed model has four hidden layers with 24 units in each one, to optimize the training process, online (stochastic) gradient descent is considered, and its results overcame the state-of-the-art approach at that time, with just a few processes. In [27], instead, the authors proposed a multi-layer perceptron (feed forward neural network) to map the input vector via two hidden layers (2000 and 1000 units each one) with the corresponding output layer having 10 units and similarly to [28], a stochastic gradient descent was used for the loss optimization.

On the other hand, in the SR field, a large number of recent contributions also based on the DL framework have been presented [24]. In this section, the most representative models based on CNN [29] are summarized. In [30], which is an extended version of [31], the authors proposed the use of Deep CNNs to restore high resolution images; this approach is referred to as the Super-Resolution Convolutional Neural Network (SRCNN). This method has a nonlinear mapping function that uses effective feature maps. The approach has three stages: patch extraction (convolution by a set of filters), non-linear mapping (high resolution patches using a 3×3 filter), and reconstruction (averaging to produce final high resolution). The SRCNN architecture has two hidden layers with 64 and 32 units, respectively, the used non-linear operator is RELU [32] and the mean square error (MSE) is used as the loss function for the training stage. Recently, ref. [33] presented a Generative Adversarial Network (GAN) with the aim of obtaining finer texture details in the restored images; the authors proposed a DL-based model termed Super-Resolution GAN (SRGAN). The main contribution was its discriminative architecture (a Residual Network: ResNet) and the loss functions. The perceptual loss function (called content loss) was the key for the generator architecture. Another deep learning-based model was presented in [3]. It was obtained as the combination of ResNet and the pixel recursive network (PixelRec). The ResNet is designed similar to the SRGAN discriminative part and the PixelRec is a feed forward convolutional network that takes a low resolution image through a series of 18–30 ResNet blocks. In these works, in addition to Peak Signal to Noise Ratio (PSNR) and Structural Similarity (SSIM) [34], human evaluations are considered, since the quality evaluation of enhanced images highly depends on human perception.

Finally, our early work based on an Artificial Neural Network (ANN) [15] mappred an RGBN color pixel to an RGB pixel value of the same scene but obtained without NIR information. The input of this network model consists of a tuple, $I=\{R_{RGBN},G_{RGBN},B_{RGBN},$$N_{RGBN}\}$, that represents a color pixel value from a single sensor camera. The model produces an output tuple, O={R,G,B}, that contains the pixel values of the image, where the NIR information has been filtered out. The green color restoration in vegetation areas, obtained by this approach, was better than using interpolation strategies; however, the restored images had smooth edge and texture regions.

## 3. Proposed Approach

**Figure 3:**
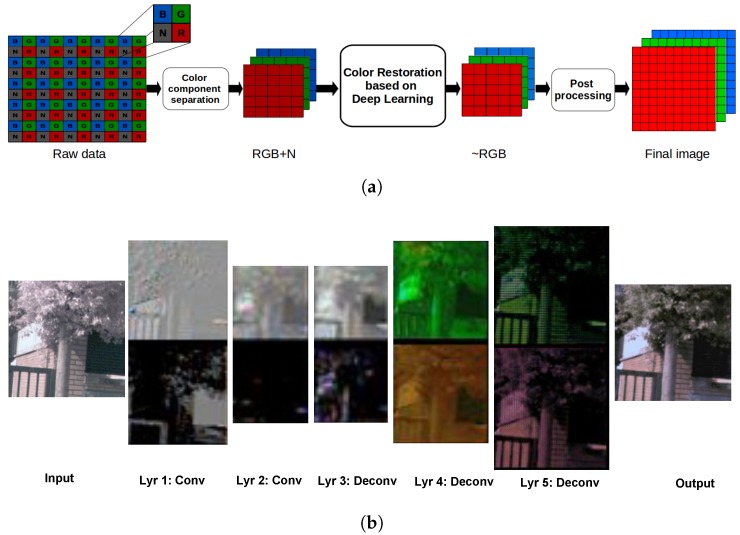
(**a**) Color restoration pipeline: the raw data corresponds to the information provided by the Single Sensor Camera (SSC); RGB+N represents the three channels affected by the NIR information which is referred to in the current work as *X*; ∼RGB is the restored color image, $\hat{Y}$; the final image is the result of the post-processing stage detailed in Section 3. (**b**) Visualization of convolutional and deconvolutional layers in ENDENet. Conv and Deconv refer to the convolutional and deconvolutional layers (Lyr: Layer).

Figure 3a shows the pipeline followed to perform the color restoration. It consisted of the following stages. Firstly, the components of the given raw image were separated into the respective channels, $I_{RGBN}=\left[R_{vis+nir},G_{vis+nir},B_{vis+nir},N_{nir}\right]$, as suggested in [11] (vis: visible spectrum band). Then, the RGB channels affected by the NIR spectral information were selected as input data ($X=[R_{vis+nir},G_{vis+nir},B_{vis+nir}]$) for the deep learning stage; the NIR channel was not considered in the current work. Next, the color restoration process was applied. Finally, the output ($\hat{Y}$) of the neural network architecture was post-processed (i.e., contrast stretch, gamma correction, and white balance). Note that after the raw mosaic data separation, the image becomes half sized; hence, to recover the full size, a bicubic interpolation was applied as a demosaicing final process. In this section, the approaches proposed for restoring RGB color in multi-spectral images are presented. Firstly, an introduction to convolutional neural network architectures is given. Then, the two CNNs proposed to restore the color information are detailed.

### 3.1. Convolutional Neural Networks

Convolution in image processing is a local linear operation in which each output pixel is the weighted sum of its neighboring input pixels [35]. For instance, for a two dimensional convolution, a squared matrix is needed, generally with an odd number dimension: (1×1), (3×3), (5×5), or (n×n). Defining the right size is key to some edge and feature detections [36,37]. This operation can be summarized as follows: $I_{conv}=I⊛W$, where *I* is the given image, ⊛ is a convolutional operation, and *W* a kernel filter.

Hence, a convolutional neural network is an Artificial Neural Network (ANN) composed of one or more convolutional layers. Each one of such layers has one or more 2D feature maps from the input image. The feature maps are obtained following the same philosophy as in the image convolution process (presented in the paragraph above). These kind of architectures are designed to extract rich information from a 2D structure. These models are easy to train and have fewer parameters than the fully connected networks [23]. In order to illustrate a CNN pipeline, Figure 3 shows convolutional layers, with the corresponding output features (see first two layers from the left). In each layer, the different features detected from the input data can be appreciated. The CNN architecture requires the setting of parameters, like strike and padding. See in Figure 3 (layers 1 and 2) how the size of each layer is smaller than previous one; this resizing is key to the detection of special characteristics sought using less hardware performance. When the aim is to have an output layer of the same size as the input one, the usage of stride-padding parameters is a solution; this is the case for image restoration. After convolutions have been performed, activation functions need to be applied to drive the network toward the target (e.g., RELU: Rectified Linear Unit [32]). In addition, another set of operations can be used to improve results, prevent overfitting, and perform an internal covariate shift to name a few (e.g., batch normalization, dropout, max-pooling).

Deconvolutional Neural Networks (DNN), which are more precisely termed transpose sparse coding convolutional networks [38], follow the same process as convolutional networksand use the same parameters as CNN. However, in the forward and backward pass, instead of extracting features and finding gradient components of the input data and the respective weights, they do so reversibly, see Figure 3 (the last three layers). This figure depicts results from the convolution and deconvolution operations. The first two layers correspond to convolution operations, where the edges and contours are extracted. The remaining deconvolutional layers reconstruct the image from the previously extracted features; note that in the output, after (the deconvolution operations), the characteristics related to the color of the image are plausibly reconstructed. As a result, we can say that the DNN generates an image from a given feature map.

Like in other kinds of models based on DL [23], either for CNN or DNN, it is necessary to prepare the data for training and to regularize and optimize the architecture [39]. In addition to the architecture, the loss function or cost function (L()) is an essential part of supervised learning. It measures the compatibility between the predictions made by the model with respect to the given ground truth. In other words, after every iteration, the computation of the loss function is needed to evaluate how well it is going through the training process. Within the context of our application (color restoration), for the minimization of L(), the Mean Square Error (MSE) is considered, which is computed as follows:

(2) $$L\left(\right)=MSE\left(Y,\hat{Y}\right)=\frac{1}{n}\sum_{i,j}^{w,h}{\left(Y_{i,j}-{\hat{Y}}_{i,j}\right)}^{2}$$

where (w,h) are the image width and image height respectively, *n* is the total number of image pixels, (i,j) are the pixel indices, $\hat{Y}$ is the value predicted by the network, and *Y* is the corresponding ground truth for the input image. Finally, the MSE value, which is used in the loss function, is used to minimize or optimize with an objective function [40].

### 3.2. Proposed Restoration Architectures

**Figure 4:**
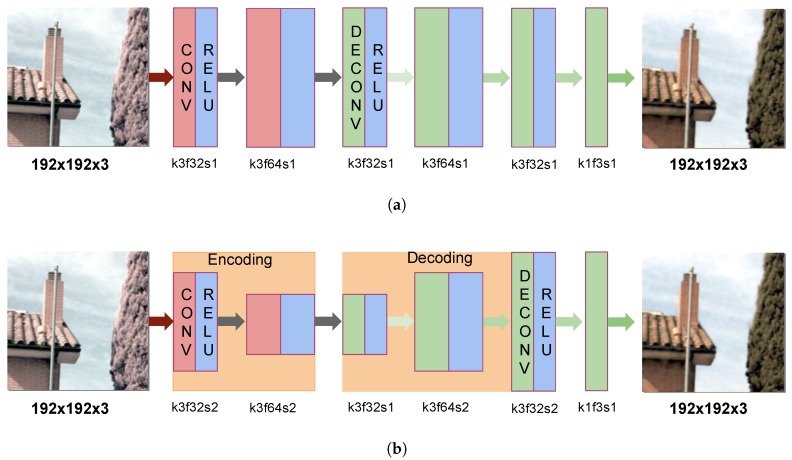
Illustration of the proposed deep learning architectures for RGB color restoration from multi-spectral images: (**a**) Convolutional and Deconvolutional Neural Network (CDNet) and (**b**) Encoder-Decoder Neural Network (ENDENet). CONV refers to convolution, DECONV to deconvolution and RELU (Rectified Linear Unit) is the non-linear function used for the layers in the respective illustration. For the term “k3f32s2”, k = kernel size (3 × 3), f = feature size (32) and s = size of stride (2, 2); the same notation is used through the illustration.

In this section, two different CNN-based architectures are proposed. The first one consists of a Convolutional and Deconvolutional Neural Network (CDNet) that is formed by two and four hidden layers, respectively (see Figure 4a). As in former work [15], CDNet attempts to clean the NIR infection in the RGB channels; therefore, in the first two convolutional layers, the input image (X) is filtered with the aim of cleaning the NIR information. Then, in the deconvolution part, it reconstructs the characteristics of the input data but without the NIR influence. Therefore, the output layer gives a predicted image $\left(\hat{Y}\right)$ supervised by the ground truth image (Y), in summary, $\hat{Y}=CDNet\left(X,Y\right)$, where $X=[R_{vis+nir},G_{vis+nir},B_{vis+nir}]$, $Y=[R_{vis},G_{vis},B_{vis}]$ and $\hat{Y}=\left[{\hat{R}}_{vis},{\hat{G}}_{vis},{\hat{B}}_{vis}\right]$. In the proposed CDNet architecture, five layers have a RELU activation function [32], and the last one is just a fully deconvolutional layer. The architecture retains the same height × width size but is not the same in terms of its number of feature maps, which are as follows: [32,64,32,64,32,3]; 64 and 32 units are used, as recommended in [30] for efficiency in regard to hardware limitations. To estimate the network parameters and minimize the loss in $\hat{Y}$, the Mean Square Error (MSE) was considered, as in the state-of-the-art techniques for restoration. Moreover, to optimize the CDNet training, AdamOptimazer with a learning rate of 0.0003 was considered. In terms of the number of layers, two convolutions followed by four deconvolutions were used; these values were obtained after evaluating different configurations with the aim of avoiding image deterioration during the convolution stages and trying to get the best result after the deconvolution stages.

In addition to the CDNet presented above, a second architecture is proposed to evaluate the capability of NIR removal from the given color infected images. This second architecture is similar to the CDNet but with down-sampling and up-sampling in the convolution and deconvolution layers. This new architecture is referred to as the Encoder-Decoder Neural Network (ENDENet). Figure 4b presents an illustration of this network and gives details of the set up of the layers. The encoder part is convolution, while the decoder is a transpose convolution (deconvolution). As depicted in Figure 4b, the first, second, fourth, and fifth layers have a (2,2) stride; hence, the image size is encoded from 192×192 to 48×48 and decoded to the same size as in the *X* size.

## 4. Experimental Results

This section presents the results obtained with the two architectures proposed in the current work. Additionally, comparisons with state-of-the-art approaches [20,21] and a super-resolution based technique, SRCNN, [30] are given.

### 4.1. System Setup

The effectiveness of the different approaches was assessed using a Single Sensor Multi-Spectral Image Dataset (SSMID) [16]. In particular, the Outdoor Multi-Spectral Image with Vegetation (OMSIV) was used. The OMSIV dataset was obtained with a SSC that captures visible and near infrared bands at the same time (see Figure 2c). Outdoor scenes with vegetation were acquired in different sunlight conditions as well as challenging environments in urban scenarios; for the purpose of the ground truth, another SSC with infrared cutoff filter was used. Then, a pair of acquired images was registered [41]. For a more detailed description of SSIMD, see [16]. Since there is a high amount of band cross-talk in images, a big volume of training data was required to train the proposed models. From the OMSIV dataset, 500 images were used for training and validation, and 32 were used for testing; the given images were 256 × 256 pixels in size. A data augmentation process was used to increase the number of patches to train both networks. From this data augmentation process 12,599 patches were obtained: 12,371 were taken for training and 128 for validating. For model evaluation purposes, the remainder 32 images were augmented to 128. A subset of 15 images is presented and analyzed in Section 4.3. During the data augmentation process, patches of (192 × 192) were obtained from the original images.

In order to obtain the best performance from the architectures presented in Section 3.2, different configurations were tested by adding and removing layers of the encoding and decoding stages [26]. Additionally, filters with sizes of (3 × 3, 5 × 5 and 7 × 7) were evaluated in both architectures in order to find the best one. The filter with a size of (3 × 3) was selected, since when large filter sizes were considered, the texture of $\hat{Y}$ was smoothed. A 1 × 1 filter was added to the last layer in ENDENet as well as CDNet. The Tensorflow library on a TITAN X GPU was considered to be the training stage. Note the MSE values are normalized on a [0–255] scale; the loss function converged to a MSE value of 50 in both models. On average, the training stage took about 6 days. The ENDENet, due to its different layer sizes, took about 1.45 min for each epoch, while the CDNet took 2 min.

### 4.2. Image Similarity Quantitative Evaluation

A key element in color restoration approaches is the similarity measure used to evaluate the performance. As mentioned in [3,30], PSNR and SSIM [42] are frequently used in color image similarity evaluations. However, in images with tiny pixelated areas or poor features, it is hard to evaluate the performance using such similarity measurements. In [3,33] in addition to these metrics, human perception evaluations were considered to quantify the ability of different approaches to perceptually reconstruct an image. In the current work, two additional metrics to PSNR and SSIM were considered, in addition to the human evaluations. They were the Angular Error (AE) and the Color Difference version 2000 (DE2000), referred to as ($\Delta E_{00}$). The AE was computed as follows:

(3) $$AE_{\left(i,j\right)}=\cos^{-1}\left(\frac{\mathrm{dot}({\hat{Y}}_{\left(i,j\right)},Y_{\left(i,j\right)})}{\mathrm{norm}\left({\hat{Y}}_{\left(i,j\right)}\right)*\mathrm{norm}\left(Y_{\left(i,j\right)}\right)}\right).$$

The AE, as for other similarity measures [42,43], is computed over every single pixel of a pair of image improving MSE values; $\hat{Y}$ is the RGB image predicted by the proposed networks, and *Y* is the corresponding ground truth image.

$\Delta E_{00}$ is computed by transforming $\hat{Y}$ and *Y* to the CIELAB color space (${\hat{Y}}_{LAB}$, $Y_{LAB}$); this color similarity measure is commonly used in the color research field, and it is a key evaluator to see the results of restoration in outdoor RGB+N images with vegetation, because, as mentioned in Section 1, the presence of NIR in vegetation desaturates the normal human color vision. For each pair of image pixels, $\Delta E_{\left(i,j\right)}$ is obtained as follows:

(4) $$\begin{array}{cc} \Delta E_{\left(i,j\right)} & =[{\left(\frac{\Delta L^{\prime }}{k_{L}S_{L}}\right)}^{2}+{\left(\frac{\Delta C^{\prime }}{k_{C}S_{C}}\right)}^{2}+{\left(\frac{\Delta H^{\prime }}{k_{H}S_{H}}\right)}^{2}\\ & +R_{T}\left(\frac{\Delta C^{\prime }}{k_{C}S_{C}}\right)\left(\frac{\Delta H^{\prime }}{k_{H}S_{H}}\right){]}^{\frac{1}{2}} \end{array}$$

where $\Delta L^{\prime }$, $\Delta C^{\prime }$ and $\Delta H^{\prime }$ are the CIELAB lightness, chroma, and hue differences, respectively; $S_{L}$, $S_{C}$, and $S_{H}$ are the weighting functions for the lightness, chroma, and hue components. $k_{L}$, $k_{C}$ and $k_{H}$ are the factors to be adjusted according to different viewing parameters; the $R_{T}$ function is intended to improve the color difference equation to fit chromatic differences in the blue region (for a detailed description see [44,45]). Once Δ$E_{\left(i,j\right)}$ was computed for the whole set of pairs of pixels, the Δ$E_{00}$ value was obtained as presented in [16]. It should be mentioned that $\Delta E_{00}$ represents the percentage of pixels with a ΔE value in between two given thresholds [a, b]. More specifically, in the current work Δ$E_{00}$ represents the percentage of pixels in the range between [0, 10]. ΔE values higher than 10 correspond to pixels with a color difference that is easily detected by human eyes (see [44] for more details). The Δ$E_{00}$ was obtained as follows:

(5) $$\Delta E_{00}=\frac{sp_{\left[a,b\right]}\times 100}{w\times h}$$

where (w,h) values correspond to the image width and image height; $sp_{\left[a,b\right]}$ is the total number of pair of pixels whose Δ$E_{\left(i,j\right)}$ values are in the range of [a,b]; it is obtained from

(6) $$\begin{array}{c} sp_{\left[a,b\right]}=0\\ sp_{\left[a,b\right]}=\sum_{i,j}^{w,h}\left\{\begin{array}{cc} sp_{\left[a,b\right]}+1 & \;\mathrm{if}\;a\leq \Delta E_{\left(i,j\right)}\leq b\\ sp_{\left[a,b\right]} & \;\mathrm{otherwise} \end{array}\right. \end{array}$$

As mentioned above, in the current work, [a, b] were set to [0, 10].

In addition to the similarity measurements presented above, qualitative evaluations were also considered over a subset of images. This subset of images was selected for human perceptual judgment and is referred to in the current work as Human Eye Perception (HEP). Basically, HEP is a survey made by different users to determine which of the images obtained by CDNet and ENDENet are the most similar to the ground truth. For this HEP test, 15 images were selected from the best (five images), the average (five images) and the worst (five images) cases, according to $\Delta E_{00}$ in the CDNet results; $\Delta E_{00}$ was selected since it is generally used in the color related literature to evaluate results by human perception. It should be mentioned that the same set of images would result if the ENDENet were considered. These 15 images were evaluated by 50 subjects. They were asked which of the images were more similar to the ground truth; in cases where images were perceptually the same, the subjects could give this answer.

### 4.3. Results and Comparisons

This section firstly details the state of the art approaches (i.e., [20,21,31]) used for comparing results with the proposed approaches. Then, the quantitative and qualitative results from the proposed approaches together with the corresponding comparisons are presented.

In [20] the authors presented three methods which were evaluated with different demosaicing algorithms and linear mappings. In the current work, following the suggestion from [20] and according to the type of sensor used in our experiments [16], a uniform RGB-NIR pattern [11] was used, then demosaicing was performed using [13], and finally, color correction was obtained through polynomial linear mapping [46], since this combination produces the best performance. The second algorithm used to compare the proposed approach was presented in [21]. This algorithm was described in Section 2. From the two MSFA patterns presented in [21], the one with best results was selected. Finally, the third comparison was performed with respect to a super-resolution CNN -ased approach [31], termed SRCNN. This super-resolution approach was selected for comparisons, since it shares similar purposes when trying to preserve features and edges in the reconstructed images. This SRCNN approach was detailed in Section 2, and the pipeline followed the same process as the proposed approaches (see Figure 3a). It should be mentioned that for comparison purposes, all resulting images were resized to the same size. This means that images resulting from SRCNN, CDNet and ENDENet, which were half the size (both width and height) of those in ref. [20,21] were up-sampled with a demosaicing technique by a bicubic interpolation to full-size (see Section 3). It should be also mentioned that similar comparative results could be obtained in the other way around, i.e., if the comparison was performed at half resolution by down-sampling results from refs. [20,21].

The dataset presented in Section 4.1 was used to evaluate the performances of the two proposed architectures as well as to compare the results with the aforementioned state-of-the art-approaches. As can be appreciated in Table 1, both network architectures produced similar results. However, when PSNR, SSIM, and AE (AE just average) metrics were considered, CDNet obtained the best results. ENDENet outperformed CDNet when just $\Delta E_{00}$ and AE (median) were considered. In all cases, the proposed architectures outperformed the three algorithms from the state-of-the art evaluated in the current work.

**Figure 5:**
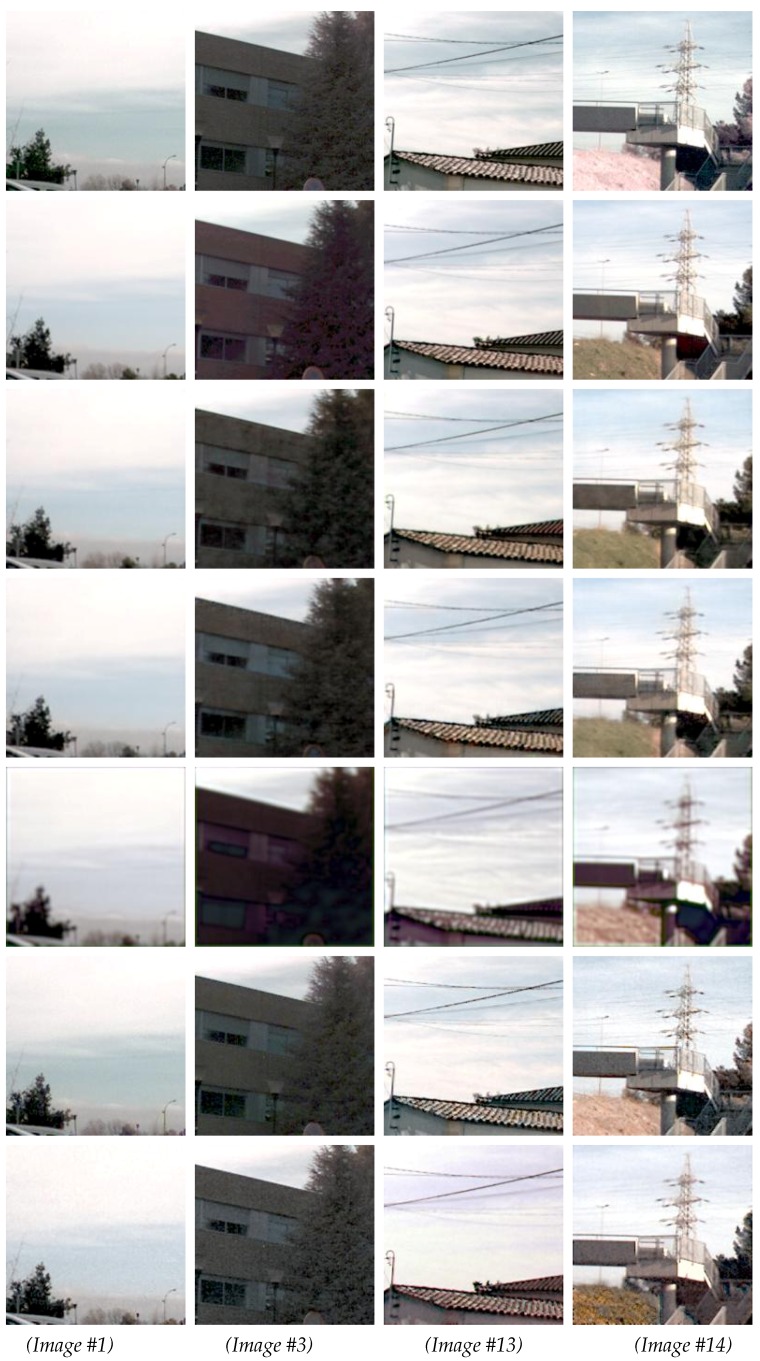
Four samples from the best results (see Table 2). (*1st row*) *X* images (RGB+N). (*2nd row*) *Y* (ground truth). (*3rd row*) Results from CDNet. (*4th row*) Results from ENDENet. (*5th row*) Results obtained with the Super-Resolution Convolutional Neural Network (SRCNN) ([30]). (*6th row*) Results obtained with ref. [21]. (*7th row*) Results obtained with ref. [20]. The image numbers correspond to the values presented in Table 2.

**Figure 6:**
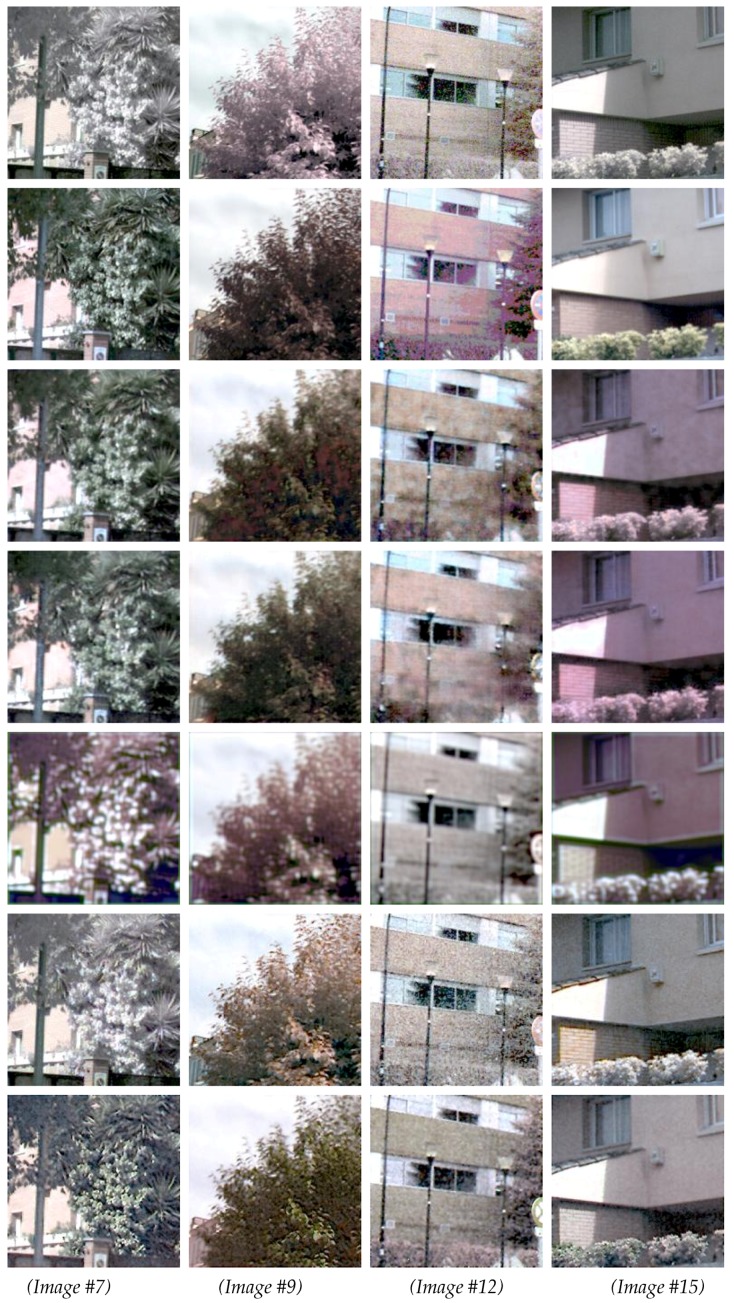
Four samples from the average and worst results (see Table 2). (*1st row*) *X* images (RGB + N). (*2nd row*) *Y* (ground truth). (*3rd row*) Results from CDNet. (*4th row*) Results from ENDENet. (*5th row*) Results obtained with SRCNN ([30]). (*6th row*) Results obtained with ref. [21]. (*7th row*) Results obtained with ref. [20]. The image numbers correspond to the values presented in Table 2.

In order to analyze the results from the two proposed architectures in more detail, a subset of 15 representative images was picked, as mentioned above. Table 2 presents results from this evaluation using the similarity metrics introduced in Section 4.2: SSIM, PSNR, AE, and $\Delta E_{00}$. On the bottom of this table, the results from the Human-Eye Perception (HEP) evaluation are depicted. The values of AE are like MSE: the smallest the better. However, for the other metrics, the largest values are better. Additionally, in the first row of each section in the table, similarity values computed between the original image (*X*) and the ground truth are provided as a reference. Figure 5 and Figure 6 present qualitative results for the images underlined in the table.

Since the images in the subset corresponded to the best, worst, and average cases, they could not be used to identify which network has the best performance. These quantitative results are just presented to appreciate the improvement with respect to the state-of-the-art methods. Actually, as can be appreciated in Table 2, ENDENet and CDNet had similar results. Taking into account the number of times a given network architecture obtained the best result. Out of the 15 images, CDNet outperformed ENDENet when the SSIM evaluation metric was considered. On the contrary, if AE was considered, the ENDENet network obtained the best results. However, in $\Delta E_{00}$, both proposed architectures had the same result. Finally, when PSNR was considered [20], CDNet and ENDENet had the same results.

As mentioned above, in addition to the quantitative evaluation, a test using 50 subjects was performed with these 15 images. From the 15 evaluations, CDNet generated the best results in eight cases, compared to ENDENet with just four cases. In the remaining three cases, both networks produced a similar result, which is consistent with the results presented in Table 1 (see detailed quantitative comparison of HED at the bottom of Table 2).

Figure 5 presents four of the images for which CDNet obtained the best results when $\Delta E_{00}$ was considered, while Figure 6 shows a pair of images with an average value and a pair of images where CDNet obtained the worst results. The last three rows in Figure 5 and Figure 6 correspond to the results obtained with [20,21,30] respectively. The four first rows correspond to the original given image (NIR infected image), ground truth, the results from the CDNet architecture, and the results from the ENDENet architecture.

As can be appreciated from Figure 5 and Figure 6, most of the images in the dataset contain vegetation. According to Table 1 and Table 2, the results from the proposed network architectures achieved the highest scores for most of the evaluation metrics. For instance, according to the median values (Table 1), more than half of $\hat{Y}$ samples obtained a $\Delta E_{00}$ value above 80%. In other words, more than 80% of pixels were restored closely to the corresponding ground truth values—their difference was almost imperceptible by the human eye. In Figure 5, Image #4 corresponds to a challenging scenario where none of the proposed approaches obtained good results. This bad result is mainly due to the materials present in the scene. More samples containing these features and materials will be required to improve the performance in cases where the $\Delta E_{00}$ is less or equal to 50%.

## 5. Conclusions and Future Work

This paper proposed two variants of a deep learning framework for RGB color restoration from multi-spectral images, specifically using images from the domain of visible and near-infrared wavelengths. The results suggest that a multi-spectral image with sufficient sunlight can be restored to the visible band keeping its main features and it will be almost imperceptible to the human eye. The proposed approaches, either ENDENet and CDNet, learn a mapping function between RGN +N to RGB without any pre-processing; these images were collected in RAW format with all of the information captured by single sensor cameras. The proposed architectures could be used to solve similar problems, such as deblurring and super-resolutions.

In future work, the proposed image restoration models will be improved by increasing the number of layers, testing other CNN-based architectures, and using the NIR channel for NIR removal into the deep learning models. Additionally, the usage of a demosaicing technique in the pre-processing stage will be considered. The hyperspectral image case will be studied by extending the approaches presented in the current work.

## Figures and Tables

**Table 1:** Average and median values of the methods tested in the current work when the whole dataset (128 samples) was considered.

Methods	PSNR		SSIM		AE		$\Delta E_{00}$	
	Average	Median	Average	Median	Average	Median	Average	Median
*X*	17.626	16.367	0.610	0.635	6.322	5.417	53.454	56.452
[20]	20.768	21.306	0.590	0.606	5.490	5.366	66.027	73.711
[21]	16.921	17.391	0.315	0.332	6.137	5.501	45.929	47.808
SRCNN [31]	18.056	17.847	0.528	0.521	7.705	6.938	50.499	51.094
ENDENet	22.544	22.894	0.690	0.691	5.338	**4.484**	**77.154**	**86.700**
CDNet	**22.874**	**22.999**	**0.704**	**0.725**	**5.237**	4.593	76.005	85.264

**Table 2:** Results for a subset of 15 images from [16] (images selected as detailed in Section 4). The underlined images are depicted in Figure 5 and Figure 6 for qualitative evaluation. Bold values correspond to the best performance in the given similarity measurement; #t corresponds to the number of times a given algorithm obtained the best performance. The last section (HEP) contains the results from the survey; each value corresponds to the number of users that selected this result as the best one. ”SAME” is used when the output from both architectures was the same for a given user.

Image #	1	2	3	4	5	6	7	8	9	10	11	12	13	14	15	
Method	PSNR															#t
*X*	26.16	11.50	25.15	16.29	16.49	13.41	15.20	18.23	12.66	15.32	16.29	16.34	25.80	17.24	15.41	-
[20]	24.99	**21.38**	**27.04**	18.76	20.89	**19.84**	19.76	22.18	18.81	**19.77**	15.31	17.86	22.07	24.63	**17.30**	5
[21]	18.70	12.68	22.70	15.17	12.69	13.50	15.88	16.60	12.64	18.90	16.57	17.78	19.24	13.87	14.06	0
SRCNN [30]	28.04	22.82	19.88	14.85	16.14	18.01	15.48	18.43	17.33	18.57	17.79	18.42	22.92	19.80	12.93	0
ENDENet	33.51	17.96	26.07	**20.31**	**23.10**	18.28	21.59	23.27	21.10	19.55	**18.84**	**19.24**	26.79	**26.28**	15.48	5
CDNet	**34.44**	15.19	26.00	20.04	23.00	18.09	**22.25**	**23.39**	**21.74**	19.62	18.63	17.88	**27.20**	26.13	15.32	5
Method	SSIM															
*X*	0.91	0.45	0.59	0.60	0.70	0.35	0.62	0.59	0.53	0.41	0.49	0.42	0.87	0.74	0.64	-
[20]	0.79	**0.70**	**0.69**	0.49	0.63	**0.51**	0.49	0.59	0.52	**0.65**	0.56	0.54	0.79	0.80	0.64	4
[21]	0.36	0.24	0.36	0.34	0.19	0.22	0.36	0.31	0.17	0.39	0.20	0.24	0.36	0.34	0.31	0
SRCNN [30]	0.87	0.67	0.52	0.36	0.49	0.45	0.37	0.50	0.54	0.60	0.58	0.44	0.72	0.70	0.48	0
ENDENet	**0.96**	0.69	0.62	0.60	0.71	0.49	0.67	**0.73**	0.58	0.62	**0.70**	**0.55**	0.85	0.84	0.65	4
CDNet	**0.96**	0.63	0.61	**0.63**	**0.73**	0.39	**0.69**	**0.73**	**0.63**	0.59	0.68	0.52	**0.87**	**0.85**	**0.67**	9
Method	AE															
*X*	1.40	5.06	8.82	3.37	3.25	9.55	6.27	8.78	4.77	5.54	4.80	7.81	1.68	4.07	4.28	-
[20]	3.55	5.28	**8.12**	4.24	3.45	6.22	8.50	8.30	7.63	**3.45**	4.02	5.89	2.72	3.09	3.83	2
[21]	1.54	5.51	9.18	3.69	3.63	**5.49**	7.95	7.79	7.96	3.96	4.50	6.67	2.74	4.34	3.71	1
SRCNN [30]	1.37	4.91	12.51	5.47	5.41	6.15	9.31	11.61	**5.52**	3.63	3.47	**5.81**	3.13	5.63	6.74	2
ENDENet	0.76	**3.76**	8.79	**3.66**	**3.38**	6.80	5.80	**6.04**	9.32	3.46	**3.03**	5.89	2.05	**2.53**	4.16	6
CDNet	**0.75**	4.13	9.14	4.02	3.76	8.07	**5.43**	6.30	6.56	4.08	3.56	6.19	**2.02**	2.71	**3.78**	4
Method	$\Delta E_{00}$															
*X*	84.52	7.70	95.34	26.44	26.64	13.38	43.92	64.98	48.51	26.85	26.40	18.52	91.64	72.96	31.71	-
[20]	99.43	**90.37**	93.14	37.54	60.69	**65.58**	71.02	80.80	75.99	48.36	15.87	16.70	58.91	88.38	**31.72**	3
[21]	30.06	11.40	90.91	23.70	14.29	21.18	53.74	59.02	22.99	37.30	11.39	16.70	46.96	28.02	26.37	0
SRCNN [30]	95.10	93.30	97.52	8.82	13.20	55.92	29.71	58.47	62.46	45.82	22.11	16.87	82.20	71.33	22.61	0
ENDENet	99.65	61.14	**93.31**	42.56	**70.24**	61.78	81.76	**90.36**	77.89	**56.63**	**52.88**	**27.27**	92.48	91.84	26.40	6
CDNet	**99.76**	36.32	92.99	**43.71**	68.14	46.65	**84.46**	87.82	**85.41**	50.55	37.81	19.27	**93.72**	**92.11**	26.56	6
Method	HEP															
SAME	**32**	8	5	9	15	8	22	16	11	13	5	6	**28**	**29**	16	3
ENDENet	13	**36**	**40**	**22**	5	10	4	15	4	4	**37**	12	11	4	12	4
CDNet	5	6	5	19	**30**	**32**	**24**	**19**	**35**	**33**	8	**32**	11	17	**22**	8
